# Effect of diminished flow in rabbit lumbar arteries on intervertebral disc matrix changes using MRI T2-mapping and histology

**DOI:** 10.1186/s12891-019-2721-y

**Published:** 2019-07-27

**Authors:** Takao Imanishi, Koji Akeda, Koichiro Murata, Akihiro Sudo

**Affiliations:** Department of Orthopaedic Surgery, Mie University Graduate School of Medicine, 2-174 Edobashi, Tsu City, Mie 514-8507 Japan

**Keywords:** *Intervertebral disc*, *Lumbar artery*, *Disc degeneration*, *MRI T2-mapping*, Rabbit

## Abstract

**Background:**

Impaired lumbar artery flow has been reported in clinical and epidemiological studies to be associated with low back pain and lumbar disc degeneration. However, it has not been experimentally demonstrated that impaired lumbar artery flow directly induces intervertebral disc (IVD) degeneration by affecting IVD matrix metabolism. The purpose of this study was to evaluate whether ligation of the lumbar artery can affect degenerative changes in the rabbit IVD.

**Methods:**

New Zealand White rabbits (*n* = 20) were used in this study. Under general anesthesia, the third and fourth lumbar arteries were double-ligated using vascular clips. The blood flow to the L3/L4 disc (cranial disc) was reduced by ligation of the third lumbar artery and that of the L5/L6 disc (caudal disc) by ligation of the fourth lumbar artery. The blood flow to the L4/L5 disc (bilateral disc) was decreased by ligation of both the third and fourth lumbar arteries. The L2/L3 disc was used as the control. Disc height was radiographically monitored biweekly until 12 weeks after surgery. The rabbits were sacrificed at 4, 8, and 12 weeks after surgery and magnetic resonance imaging (MRI) T2-mapping, histology and immunohistochemistry were assessed.

**Results:**

Lumbar artery ligation did not induce significant changes in disc height between control and ischemic discs (cranial, bilateral and caudal discs) during the 12-week experimental period. T2-values of ischemic discs had no significant trend to be lower than those of the control L2/L3 discs. Histologically, Safranin-O staining changed following ligation of corresponding IVD lumbar arteries. Histological grading scores for disc degeneration, which correlated significantly with MRI T2-values, had significant changes after the surgery. Immunohistochemical analysis showed that the ligation of lumbar arteries significantly affected a change in the percentage of HIF-1α immunoreactive cells of ischemia discs compared to that of control discs four weeks after the surgery (*p* < 0.05).

**Conclusions:**

The MRI and histology results suggest that diminished flow in lumbar arteries induce mild changes in the extracellular matrix metabolism of rabbit IVDs. These matrix changes, however, were not progressive and differed from the degenerative disc changes seen in the process of human IVD degeneration.

## Background

Low back pain is a musculoskeletal condition prevalent in the aging population that is associated with work-related disabilities and reduced quality of life [1]. Intervertebral disc (IVD) degeneration, clinically characterized by radiographic findings including the decreased signal intensity of magnetic resonance imaging (MRI) T2-weighted imaging [2] and disc height narrowing [3], is a proven major contributor to discogenic low back pain [4]. IVD degeneration is thought to result from micro-environmental changes influenced by multiple contributing factors, including aging, sex, predisposing injury, genetics, and environment [5–7]. The exact mechanism of IVD degeneration has yet to be fully elucidated.

The IVD is anatomically classified as a symphysis. It consists of the gelatinous nucleus pulposus (NP) surrounded by the annulus fibrosus (AF), a concentrically organized lamella structure of collagen fibers. The NP is rich in an extracellular matrix (ECM) that mainly contains the hydrophilic proteoglycan (PG) “aggrecan” attached to many glycosaminoglycan (GAG) chains. A definitive biochemical feature of IVD degeneration is degradation of the ECM resulting from loss of PGs and collagens caused by the homeostatic imbalance between anabolism and catabolism [7].

Blood flow to the vertebral bodies of the lumbar spine is abundantly supplied by the lumbar arteries, branches of the abdominal aorta [8]. However, the IVD is an avascular structure, except for the outer-most layer of the AF [9]. Essential nutrients, such as oxygen and glucose, are supplied to the discs by capillaries that arise in the vertebral bodies and penetrate the subchondral bone (see review in [10]). These nutrients are then transported to IVD cells mainly by diffusion through the dense ECM [10]. The microenvironment of the center of the IVD is considered, therefore, to have the lowest concentration of glucose and oxygen [11].

Recent evidence shows that survival of IVD cells, particularly those of the NP, in an environment of hypoxia and low glucose, is associated with expression of hypoxia inducible factor (HIF)-1α. HIF is essential in the maintenance of anaerobic-glycolysis and cellular response to the hypoxic conditions of nutrient stress.

Because nutrient transport into IVD tissues is problematic, especially into the center of the NP, interruptions in the supply of nutrients have been found to be strongly associated with the progression of IVD degeneration [10].

Both clinical and epidemiological studies have indeed shown that impaired flow due to atherosclerosis of the lumbar arteries is associated with lumbar disc degeneration and low back pain (see review in [12]). No in vivo animal study has demonstrated that impaired flow in the lumbar arteries directly induces IVD degeneration. From results of previous studies [12–17], we hypothesized that factors that reduce the blood supply to the lumbar spine can cause micro-environmental changes within lumbar IVDs, leading to degenerative changes in IVDs.

The purpose of this study is to determine the effect of ischemia of lumbar vertebra on disc height and ECM changes using a rabbit lumbar artery ligation model.

## Methods

### Rabbit lumbar artery ligation model

This study was carried out in strict accordance with the recommendations in the Guide for the Care and Use of Laboratory Animals of the National Institutes of Health. The protocol of this study was approved by the Institutional Animal Care and Use Committee of Mie University (reference number: 21–11). Twenty-two 12-week-old New Zealand White rabbits (female) weighing 2.4–3.0 kg (Kitayama Labs Laboratory Animals Bleeding & Equipment Supply, Ina, Japan) were housed in separate cages under standard conditions with a light-dark cycle (12 h – 12 h) and dry-bulb room temperature at 22–24 (°C), and provided ad libitum access to tap water and food pellets daily.

**Fig. 1:**
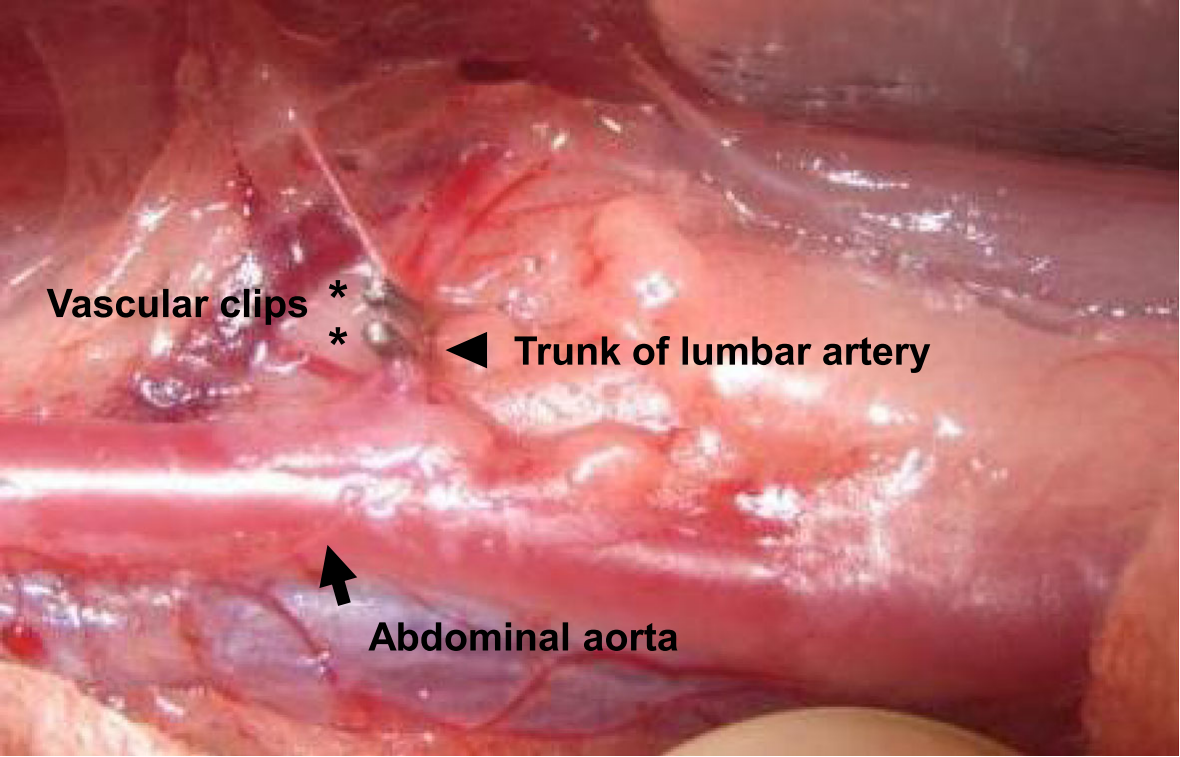
Intraoperative picture of rabbit lumbar artery ligation. The abdominal aorta (arrow) was exposed by a retroperitoneal approach. The trunk of the lumbar artery (arrowhead) was doubly ligated using vascular clips (asterisk)

**Fig. 2:**
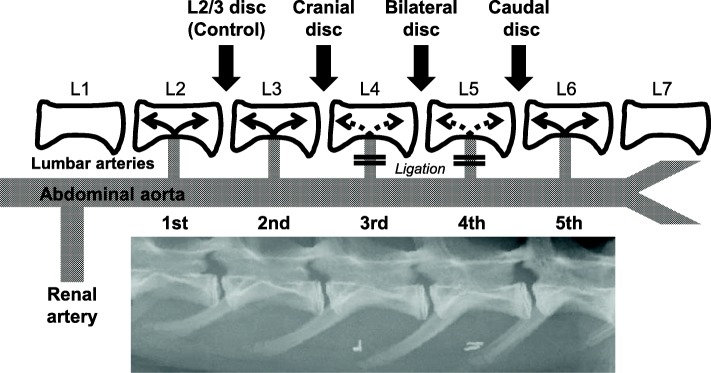
The rabbit lumbar artery ligation model. Blood flow to the L3/L4 disc (cranial disc) was diminished by ligation of the third lumbar artery, and blood flow to the L5/L6 disc (caudal disc) was diminished by ligation of the fourth lumbar artery. The L4/L5 disc was termed the bilateral disc because blood flow to the L4/L5 disc was diminished by ligation of both the third and fourth lumbar arteries. The L2/L3 disc was used as the control disc

Following intramuscular administration of 25 mg/kg ketamine hydrochloride (Ketalar®, Daiichi Sankyo, Tokyo, Japan) and 5 mg/kg xylazine hydrochloride (Selactar®, Bayer Yakuhin, Tokyo, Japan), lateral plain radiographs were taken to determine preoperative baseline values for IVD height. After sedation and induction, anesthesia was maintained with 3.0% isoflurane (Schering-Plough Animal Health, Tokyo, Japan) using anesthesia mask (Acoma medical, Tokyo, Japan). The rabbits were placed in a lateral prone position, and a posterolateral retroperitoneal approach exposed the abdominal aorta from the left renal artery to the aortic bifurcation. Lumbar arteries L2-L6 were isolated, and the third and fourth lumbar arteries providing blood flow to L4 and L5 vertebrae were doubly ligated using vascular clips (Ligaclip®, Ethicon, Somerville, NJ, USA) (Fig. 1). The disruption of blood flow (collapse of lumbar arteries) just distal to the ligation site was confirmed under direct view through a magnifying glass (× 2.9 magnification, Surgical Acuity™, Middleton, WI, USA) in all the rabbits. Blood flow to the L3/L4 disc (cranial disc) was diminished by ligation of the third lumbar artery, and blood flow to the L5/L6 disc (caudal disc) was diminished by ligation of the fourth lumbar artery. The L4/L5 disc was termed the bilateral disc because blood flow to the L4/L5 disc was diminished by ligation of both the third and fourth lumbar arteries. The L2/L3 disc was used as the control disc (Fig. 2). The wound was closed with layered sutures. The rabbits were returned to their cages after recovery observation.

Among seventeen rabbits that operated, two rabbits showed paralysis of the hind legs immediately after the surgery and not included in the experiment. These two rabbits were euthanized by carbon dioxide inhalation following premedication by the intramuscular administration of ketamine hydrochloride (25 mg/kg). 100% carbon dioxide gas was introduced into the chamber where the rabbits were placed (with its fill rate at approximately 10 to 30% of the chamber volume per minute).

The remaining fifteen rabbits showed no abnormality movement of the hind legs, and showed similar body weight changes throughout the experimental period.

Under general anesthesia induced by the intramuscular administration of ketamine hydrochloride (25 mg/kg) and xylazine hydrochloride (5 mg/kg), lateral plain radiographs of the lumbar spine were taken before surgery and at 2, 4, 6, 8, 10, and 12 weeks after surgery. At 4, 8, and 12 weeks after surgery, five rabbits per group were euthanized as described above. Intact spinal columns were harvested for magnetic resonance imaging (MRI) and histological analyses to determine the degree of degeneration. Five 12-week-old rabbits with a sham operation that only exposed the abdominal aorta were used for a 0-week control group.

### Radiographic analysis

**Fig. 3:**
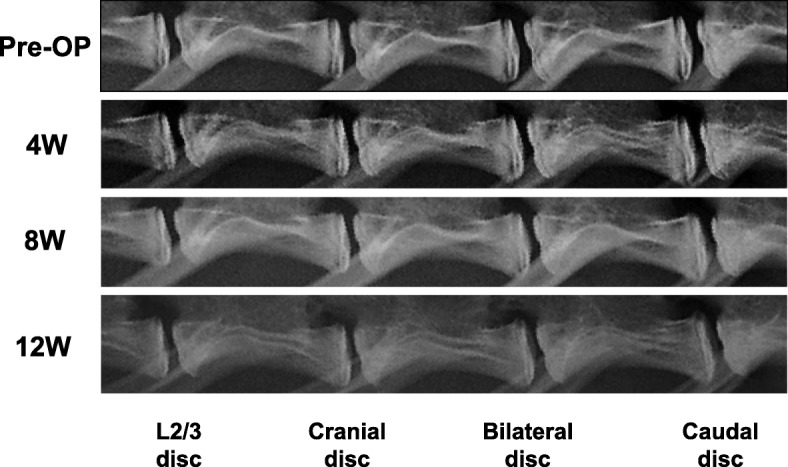
Representative lateral radiographs of rabbit lumbar spines. Representative lateral radiographs of rabbit lumbar spines at pre-operation (pre-OP) and 4, 8, and 12 weeks after lumbar artery ligation surgery are shown

Disc height was radiographically monitored biweekly from the day of operation to 12-weeks post-operation (Fig. 3). The soft X-ray radiograms were digitized and measurements of vertebral body height and disc height using OsiriX Imaging Software (OsiriX Foundation, Geneva, Switzerland) were made by an orthopedic researcher blinded to the treatment group. Intervertebral disc height was expressed as the disc height index (DHI), as previously described [18]. A change in the DHI for a disc was expressed as percent disc height index (%DHI) and normalized to the measured preoperative disc height (%DHI = [postoperative DHI/ preoperative DHI] × 100).

### MRI T2-quantification

After the rabbits were sacrificed, the L1 to L6 vertebrae with surrounding soft tissues were isolated and wrapped by plastic cling film to prevent dehydration, and kept in ice box until subjecting to quantitative transverse relaxation time (T2) MRI analysis as previously reported [19]. MRI was performed using a 3.0-Tesla imager (Achieva 3.0 T, PHILIPS, Amsterdam, The Netherlands) with a 3″ birdcage extremity coil (PHILIPS). Room temperature was kept constant at 24 °C during the MRI scan. T2 mapping was performed using a multi-echo spin-echo sequence in the sagittal plane. Scanning parameters were: Repetition Time (TR) = 3000 ms, Echo time (TE) = 20, 40, 400 ms (20 TEs), field of view = 10 cm, slice thickness = 2 mm, image matrix = 560 × 560, number of excitation = 1. Total scanning time per sample was 10 min 39 s. For creating color-coded T2-maps, MR images at multiple TE were imported into OsiriX Medical Image software by a T2 mapping plug-in.

**Fig. 4:**
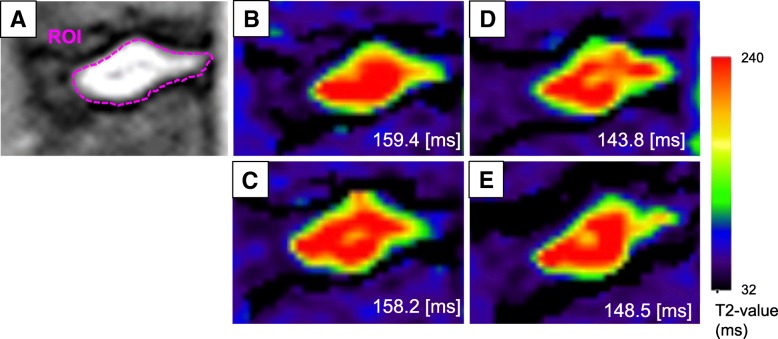
Representative color-coded T2 maps in the sagittal plane of the rabbit lumbar spine. The region of interest (ROI) was determined at the borders of the annulus fibrosus (AF) and nucleus pulposus (NP) (**a**). At eight weeks after surgery, compared to the control disc (**b**), the signal intensity in the center of the nucleus pulposus changed in the cranial disc (**c**), bilateral disc (**d**) and caudal disc (**e**). T2-value (ms) of each disc was indicated

MRI analysis was performed on IVDs L2/L3, L3/L4, L4/L5 and L5/L6. Mean signal intensities were determined in the regions of interest (ROI) framing the inner border of the AF on T2-weighted images taken at TE of 100 ms (Fig. 4A). Images were selected that best visualized the borders of the AF and NP (Fig. 4B-E).

### Histological analysis

Intervertebral disc samples were harvested at 4, 8, and 12 weeks after surgery to analyze the degree of degeneration over time. After MRI assessment, the experimental IVDs were fixed in 4% formalin, soaked in a decalcifying agent (K-CX: FALMA, Tokyo, Japan), embedded in paraffin, and sectioned midsagittally. The samples were stained with hematoxylin and eosin for analysis of cellular constituents and Safranin-O for PG content. An observer, blinded to the experiment, analyzed the histological sections and graded them using a previously reported protocol [18, 20]. Histological grading of midsagittal sections was performed based on a scale using four categories of degenerative change, including (A) patterns of fibrocartilage in the lamellae of the AF, (B) the border between the AF and NP, (C) the cellularity of the NP, and (D) the matrix of the NP, as well as (E) the histological changes of the pericellular matrix of NP cells (Table 1). Discs were graded with a value from 1 to 3 in each of the five parameters. A normal disc was assigned a value of 1 and a degenerated disc was assigned a value of 3. Grades ranged from 5 to 15 with a maximum of 3 points for each parameter; therefore, a grade of 15 represents severe degeneration. The total scores for each disc in this semi-quantitative grading assessment were statistically analyzed.

**Table 1:** Definition of a Histological Grading Scale

A. Annulus fibrosus	Grade
Normal, pattern of fibrocartilage lamellae (U-shaped in the posterior aspect and slightly convex in the anterior aspect) without ruptured fibers and without a serpentine appearance anywhere within the annulus	1
Ruptured or serpentined patterned fibers in less than 30% of the annulus	2
Ruptured or serpentined patterned fibers in more than 30% of the annulus	3
B. Border between the annulus fibrosus and nucleus pulposus	
Normal	1
Minimally interrupted	2
Moderate / severe interruption	3
C. Cellularity of the nucleus pulposus	
Normal cellularity with large vacuoles in the gelatinous structure of the matrix	1
Slight decrease in the number of cells and fewer vacuoles	2
Moderate / severe decrease (> 50%) in the number of cells and no vacuoles	3
D. Matrix of the nucleus pulposus	
Normal gelatinous appearance	1
Slight condensation of the extracellular matrix	2
Moderate / severe condensation of the extracellular matrix	3
E. Pericellular matrix of the nucleus pulposus	
Normal	1
Slight formation of the pericellular matrix	2
Moderate / severe formation of the pericellular matrix	3

Semi-Quantitative histological analysis was performed using a grading scale based on four categories of degenerative changes (A-D) [18, 20]. The pericellular matrix of the nucleus pulposus (E) was also histologically graded

### Immunohistochemical analysis

IVD samples (L2/L3 to L5/L6) of five rabbits at 4 weeks after surgery were representatively used for immunohistochemical analysis. Five L2/L3 discs at 4 weeks after surgery were used as the non-ischemic control. Samples were fixed, decalcified, and embedded in paraffin for serial 5 μm sectioning used for immunohistochemical analysis. Following endogenous peroxidase inactivation and heat-induced epitope retrieval, the sections were stained with anti-HIF-1α antibody (H1alpha67: Novus Biologicals, Littleton CO, USA) for immunohistochemical analyses. Mouse IgG (DakoCytomation, Glostrup, Denmark) was used as the isotype or negative control. The sections were visualized using the universal immuno-enzyme polymer method (Histofine Simple Stain MAX-PO; Nichirei Biosciences, Tokyo, Japan) and 3,3′-diaminobenzidine tetrahydrochloride (DAB; Dojindo, Tokyo, Japan), followed by counterstaining with Mayer’s hematoxylin.

The number of immunoreactive cells was counted by one observer who was blinded to the experimental group. The percentage of positive-staining cells in the outer AF (oAF), inner AF (iAF) and NP area was quantified using the mean percentage of immunopositive cells from five fields of microscopic images per sample at 200x magnification.

### Statistical analysis

Differences in %DHI and MRI T2-values were assessed for statistical significance by two-way repeated measures analysis of variance (ANOVA) to compare the IVD levels with time points of examination, followed by the Bonferroni post hoc test. Sample size was determined with a power analysis for an alpha of 0.05 and power of 0.80 using G*POWER3 [21] with a sample size of 5 animals at each time point. Histological grading scores were assessed by the Kruskal-Wallis test for inter-group comparisons and the Friedman test for temporal changes. The correlation between MRI T2-values and histological grading scores was evaluated using Spearman’s rank-order correlation test. The % of immuno-positive cells was evaluated by two-way ANOVA to compare IVD levels and disc area, followed by the Bonferroni post hoc test. All data were expressed as mean ± standard deviation (SD). All the statistical analyses were performed using IBM Statistical Package for Social Sciences Software (SPSS) Statistics (IBM Japan, Tokyo). The accepted level of significance was *p* < 0.05.

## Results

### Change in disc height

**Fig. 5:**
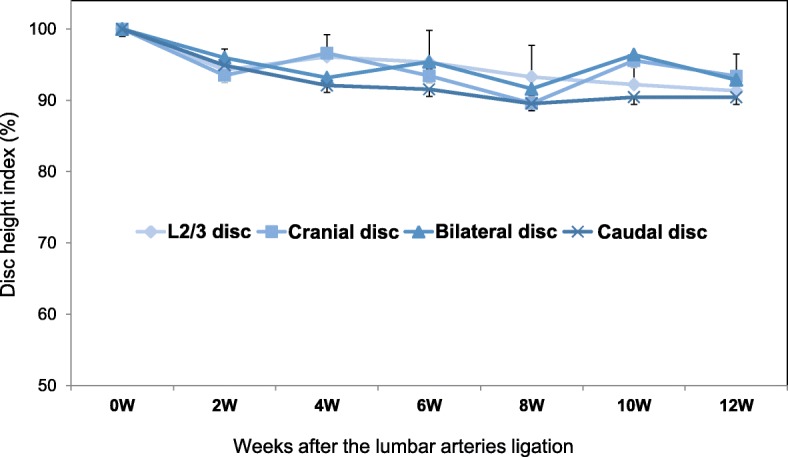
Change in percent disc height index (%DHI). No significant difference in %DHI was found between the L2/L3 (control) discs and the ischemia group (cranial, bilateral and caudal discs) in the rabbit lumbar ligation model

Representative radiographs before and after lumbar artery ligation showed no remarkable narrowing of cranial, bilateral or caudal discs compared to before surgery and the intact L2/L3 disc of the same animal (Fig. 5). The %DHI of each experimental group significantly decreased during the observation period (*p* < 0.01, 2-way repeated measures ANOVA), although the analysis of variance showed no significant interaction between disc level and time-point (*p* = 0.83). The %DHI at each disc level changed similarly during the observation period and no significant differences were found among the disc levels (Fig. 5). No significant differences were also seen in the %DHI among the four groups at each time point (Fig. 5).

### MRI assessment

Representative T2-mapping images of control (L2/L3) discs at 8 weeks postoperative showed that higher T2 values, as indicated by red pixels, were uniformly observed in the NP of IVDs (Fig. 4B). However, the loss of red pixels, replaced by yellow pixels, was identified in the center of the NP of cranial, bilateral, and caudal discs (Fig. 4C-E).

T2-values of the control, cranial, bilateral, and caudal discs (*n* = 5, respectively at each time point) showed significant decreases during the experimental period (*p* < 0.01, 2-way repeated measures ANOVA), although the analysis of variance showed no significant interaction between disc level and time-point (*p* = 0.58). The averaged T2-values of the ischemia group of cranial, bilateral and caudal discs were lower than that of the control L2/L3 group throughout the observation period but failed to reach statistical significance (*p* = 0.2, 2-way repeated measures ANOVA). No significant differences in T2-value were also found among the ischemia group of cranial, bilateral and caudal discs.

**Fig. 6:**
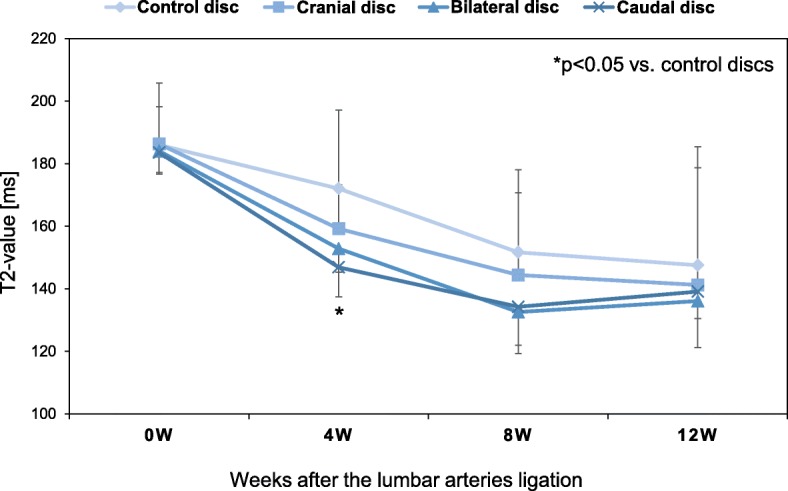
Magnetic resonance imaging (MRI) T2 values. Magnetic resonance imaging (MRI) analyses were performed at 0 (sham-operated), 4, 8 and 12 weeks after rabbit lumbar artery ligation surgery. A significant reduction in T2-values of cranial discs was observed at 4 weeks after surgery compared to the control (L2/L3) disc (*p* < 0.05)

When the data were analyzed at each time point, the average T2-value of the cranial group was significantly lower than that of the L2/L3 control group at 4 weeks postoperative (T2-values [ms]: control: 172.1 ± 10.0, cranial: 159.2 ± 5.6, bilateral: 152.8 ± 7.5, caudal: 146.9 ± 9.5, *p* < 0.05 vs. control) (Fig. 6). No significant differences were seen in T2-values among the four groups at 0, 8, 12 weeks after surgery (Fig. 6).

### Histological assessment

**Fig. 7:**
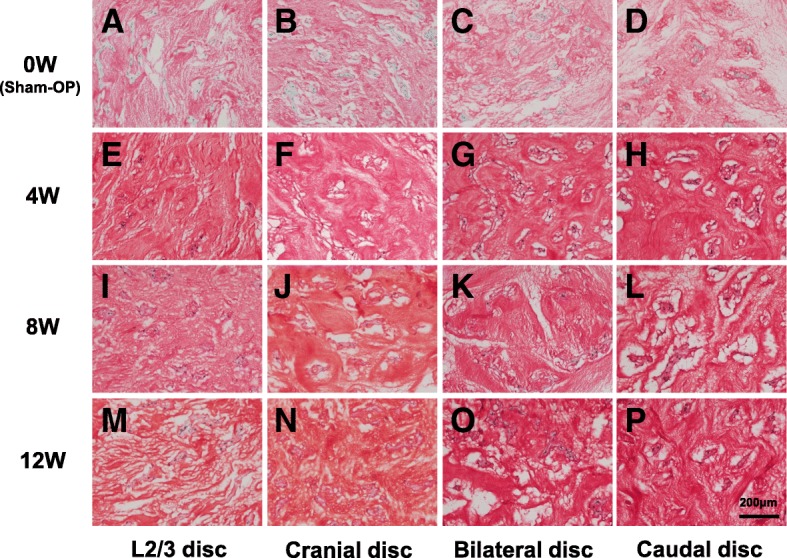
Histology of the rabbit nucleus pulposus (NP) after lumbar artery ligation. Notochordal cells were embedded in a gelatinous matrix that was uniformly stained with Safranin-O in the NP of sham-operated rabbits (Sham-OP) (0 W) discs (**a**, **b**, **c**, **d**). Safranin-O staining properties changed after ligation of lumbar arteries. At 4 weeks (**f**, **g**, **h**) and 8 weeks (**j**, **k**, **l**), the staining patterns were irregular, and intense staining is seen in the pericellular matrix. At 12 weeks (N, O, P), an irregular staining pattern remained. L2/L3 discs (**e**, **i**, **m**) were used as controls

**Fig. 8:**
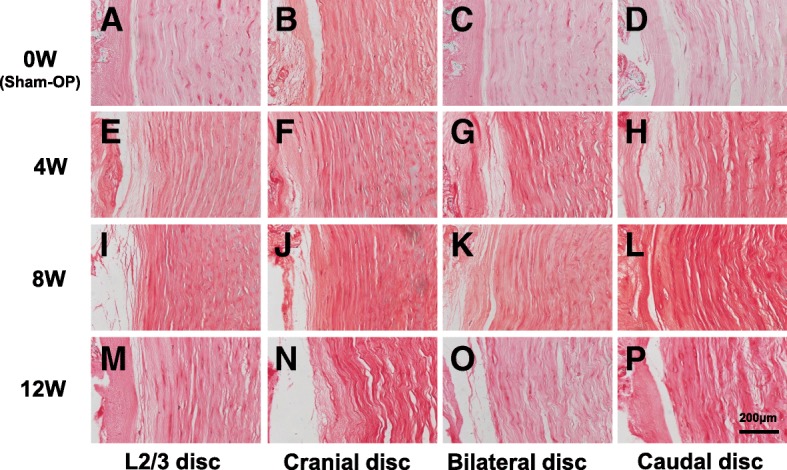
Histology of the rabbit annulus fibrosus (AF) after lumbar artery ligation. Fibrochondrocyte-like cells were aligned longitudinally between the collagen fiber bundles stained with Safranin-O in the AF of sham-operated rabbits (Sham-OP) (0 W) discs (**a**, **b**, **c**, **d**). No remarkable changes in the pattern of fibrocartilage lamellae and serpentine pattern of fibers were found at 4 weeks (**f**, **g**, **h**), 8 weeks (**j**, **k**, **l**) and 12 weeks (**n**, **o**, **p**) after ligation of lumbar arteries. L2/L3 discs (**e**, **i**, **m**) were used as controls

Histological changes were observed in NP tissues of cranial, bilateral, and caudal discs (*n* = 5, respectively at each time point) after lumbar artery ligation (Fig. 7); however no histological changes were found in AF tissues (Fig. 8). At 4 weeks post-ligation, the Safranin-O staining properties of the pericellular matrix of ischemic cranial, bilateral and caudal discs had become irregular (Fig. 7F-H). Circumferential condensation of the ECM, as shown by intense staining for Safranin-O in/around the pericellular matrix, was found in the NP of cranial, bilateral and caudal disc tissues (Fig. 7F-H). At 8 weeks, an irregular and circumferential staining pattern of the pericellular matrix was found in NP tissues of cranial, bilateral and caudal discs similar to those at 4 weeks post-ligation (Fig. 7J-L). At 12 weeks, diffuse staining for Safranin-O was seen in both the pericellular and interstitial matrix in the NP of cranial, bilateral and caudal discs (Fig. 7N-P).

In AF tissues, fibrochondrocyte-like cells were aligned longitudinally between the collagen fiber bundles. However, no remarkable changes in the pattern of fibrocartilage lamellae and serpentine pattern of fibers were found (Fig. 8). No ruptured fibers were identified in the AF tissues.

**Fig. 9:**
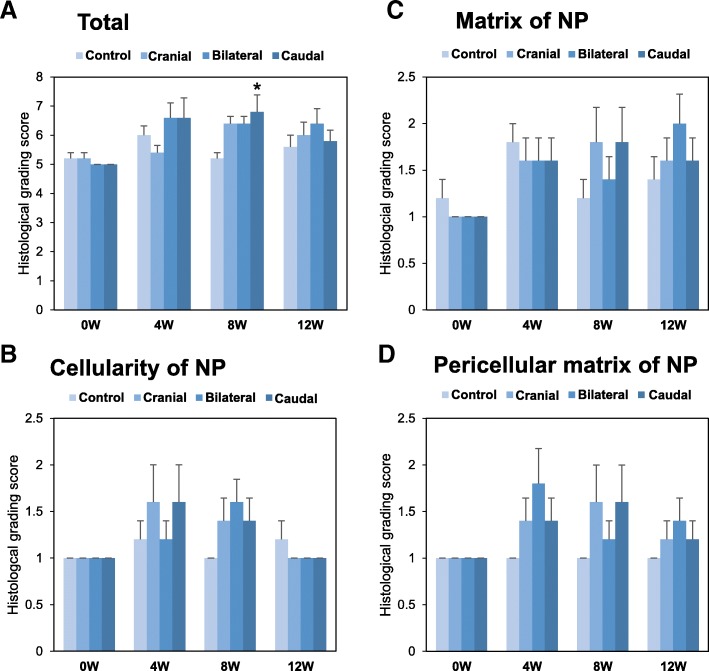
Histological grading scores. Histological grading scores for the total of five categories: (**a**), nucleus pulposus (NP) cellularity (**b**), NP matrix (**c**) and pericellular matrix of the NP (**d**) in the caudal disc, bilateral disc and cranial disc of sham-operation (0 W) and 4 W, 8 W and 12 W after lumbar artery ligation. *p < 0.05 vs. the control discs

The total histological grading scores showed that no significant differences among the four groups (*n* = 5) were seen at 0, 4 and 12 weeks post-surgery (Fig. 9A). However, lumbar artery ligation did significantly affect the total histological score of corresponding IVDs at 8 weeks after the surgery (*p* < 0.05, Kruskal-Wallis test) (Fig. 9A). The total scores for cranial disc were significantly increased compared to those of the L2/L3 control disc (p < 0.05, Fig. 9A).

When changes in total histological scores over time were analyzed using the Friedman test, lumbar artery ligation had a significant (p < 0.05) effect on temporal changes of scores of ischemic, but not of control (L2/L3) discs. Compared to 0-week discs, total histological scores of caudal discs were significantly higher at 4 weeks after surgery and those of cranial, bilateral and caudal discs were significantly higher at 8 weeks after surgery. However, for all ischemic discs, no significant differences were identified at 12 weeks after surgery compared to those of 0 weeks.

When histological grading scores were evaluated for each category, the scores of the AF (category A) and the border between the AF and NP (category B) were constant at grade 1 in all discs and did not change throughout the experimental period. The histological grade of other categories including NP cellularity (category B, Fig. 9B), NP matrix (category C, Fig. 9C) and NP pericellular matrix (category D, Fig. 9D) showed no significant differences in both the inter-group comparisons and temporal changes.

### Correlation between MRI T2-values and histological grading scores

**Fig. 10:**
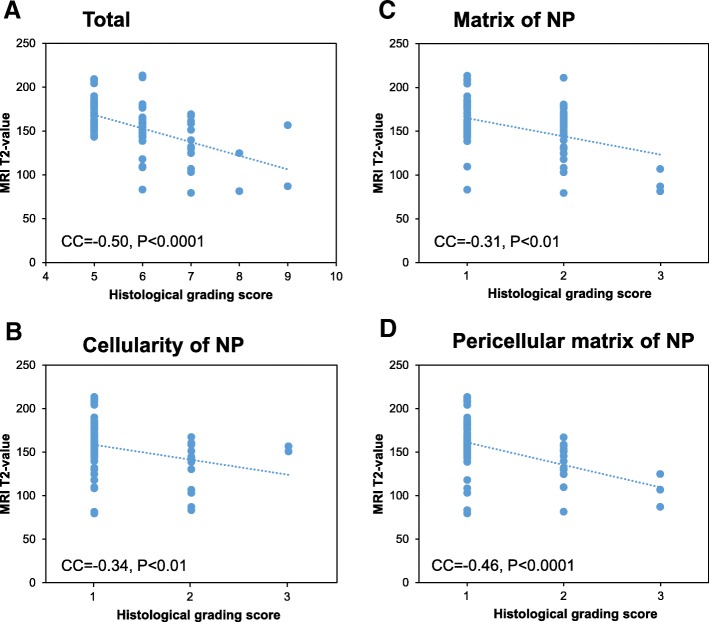
Correlation between MRI T2-values and histological scores. Correlation between the MRI T2-values and histological grading scores of the total of all five categories (**a**), nucleus pulposus (NP) cellularity (**b**), NP matrix (**c**) and NP pericellular matrix (**d**). Correlation coefficient: CC

There was a significant negative correlation between MRI T2-values and total histological grading scores (correlation coefficient [CC] = − 0.50, *p* < 0.0001, Spearman’s rank-order correlation test, Fig. 10A). A significant negative correlation was also identified between MRI T2-value and every histological parameter of NP tissues (NP cellularity: CC = − 0.34, *p* < 0.01; NP matrix: CC = − 0.31, p < 0.01; NP pericellular matrix: CC = − 0.46, p < 0.0001) (Fig. 10B-D).

### Immunohistological analysis of HIF-1α

**Fig. 11:**
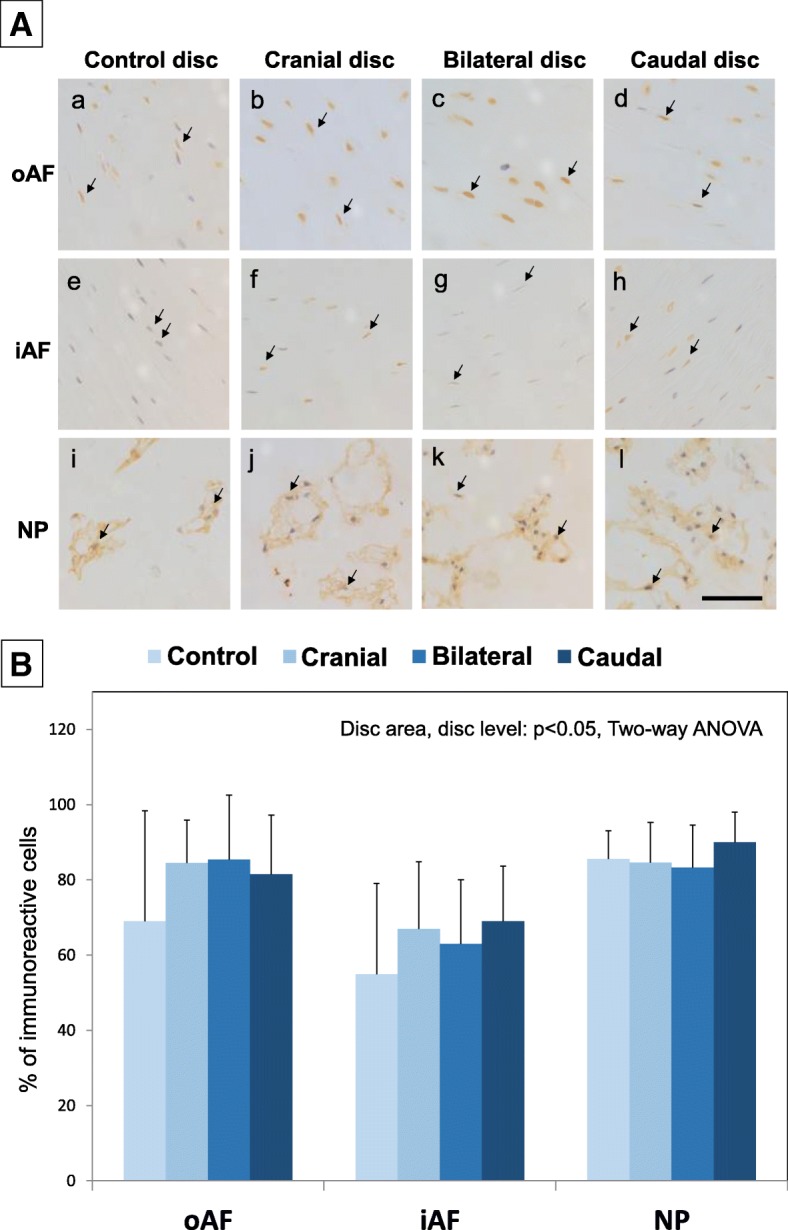
Immunochemical analyses of hypoxia inducible factor (HIF)-1α in rabbit intervertebral discs four weeks after lumbar artery ligation. A: HIF-1α immunoreactivity was clearly detected in the nuclei and cytoplasm of the cells in the outer AF (oAF) (**a**, **b**, **c**, **d**), inner AF (iAF) (**e**, **f**, **g**, **h**) and nucleus pulpous (NP) (**i**, **j**, **k**, **l**) of both control (L2/3) (a, e, i) and ischemia (cranial (**b**, **f**, **j**), bilateral (**c**, **g**, **k**) and caudal (**d**, **h**, **l**)) discs. Bar: 100 μm. B: Percentage of HIF-1α immunoreactive cells in outer AF (oAF), inner AF (iAF) and nucleus pulpous (NP) of both control (L2/3) and ischemia (caudal, bilateral and cranial) discs. Two-way analysis of variance (ANOVA) revealed that the percentage of HIF-1α immunoreactive cells was significantly associated both with disc area (oAF, iAF, NP) and disc level (control and ischemia discs) at 4 weeks after the ligation of lumbar arteries (*p* < 0.05)

HIF-1α immunoreactivity was clearly detected in the nuclei and cytoplasm in the AF and NP cells of both control and ischemia (cranial, bilateral and caudal) discs (Fig. 11A). Statistical analysis revealed that the percentage of HIF-1α immunoreactive cells was significantly associated both with disc area (oAF, iAF, NP) and disc level (control and ischemia discs) at 4 weeks after the ligation of lumbar arteries (*p* < 0.05). Analysis of variance also showed a significant interaction between the disc area and disc level (p < 0.05). A post-hoc test revealed that the percentage of HIF-1α immunoreactive cells in the NP was significantly higher than that of the iAF (NP: 85.9 ± 9.6%, iAF: 63.5 ± 19.2%, oAF: 81.2 ± 19.8%, p < 0.01, between NP and iAF) (Fig. 11B). The percentage of immunoreactive cells of caudal discs in total area (oAF, iAF and NP) was significantly higher than that of control (L2/3) discs (control discs: 71.3 ± 25.4%, cranial discs: 78.7 ± 15.8%, bilateral discs: 77.2 ± 18.3%, caudal discs: 80.2 ± 15.6%, p < 0.05 between control and caudal discs) (Fig. 11B).

## Discussion

The effect of ischemia of lumbar vertebrae on degenerative changes in a rabbit lumbar artery ligation model was evaluated in this study using radiographic disc height measurement and ECM changes of corresponding IVDs for this assessment. Our results indicate that lumbar artery ligation induced no significant change in disc height over the experimental period compared to control discs. On the other hand, MRI T2-values in the NP of corresponding IVDs showed a tendency to decrease compared to control discs. Histological analyses showed changes in Safranin-O staining properties of corresponding IVDs following lumbar artery ligation.

Several experimental animal models of the process of IVD degeneration have been developed, although no animal model that completely mimics human IVD degeneration currently exists [22]. Hou and colleagues recently reported a rabbit model for ischemic lumbar vertebrae induced by injection of pingyangmycin into vertebrae adjacent to the endplate and found that IVD degeneration was actually induced in this model [23]. Importantly, they also examined the anatomy of rabbit lumbar arteries using digital subtraction angiography (DSA) and vascular cast, and reported that, differing from human anatomy, the trunk of the lumbar arteries started directly from the aorta, divided into right and left branches that subdivided into internal and external branches going to several areas of the lumbar spine [23]. Therefore, in our animal model, blood flow of the third and fourth lumbar arteries would be almost completely impaired by double ligation of those trunks using vascular clips (Fig. 1). Our model using ligation of rabbit lumbar arteries could be thought of as a vertebral ischemia model impairing blood flow to the lumbar spine more proximally than the model reported by Hou et al. [23].

Transverse relaxation time (T2) mapping has been shown to quantitatively evaluate changes in molecular composition and structural integrity of IVDs [24–26]. Previous studies have shown significant correlation between T2 values, water and PG content and arrangement of the collagen network structure [24, 27]. The NP, playing a role as an internal semi-fluid mass, is rich in the large, negatively-charged proteoglycans (PGs) [28]. The PGs consist of a core protein that is covalently attached to glycosaminoglycan (GAG) chains, which are responsible for a water-retaining property of the NP. Therefore, T2-value of the NP reflects water concentration, which is also associated with the biochemical function of extracellular matrix molecules such as large PGs “aggrecan.”

T2-values in human IVDs have been shown to be significantly correlated with the stage of disc degeneration [24–26]. Recent studies have reported that this MRI technique can detect matrix changes in the very early stages of disc degeneration [28, 29] and from physiological loading [30]. The results of our T2-mapping evaluation indicated that the T2-value had decreased in the center of NP tissues of the ischemia group. Because the microenvironment in the center of the NP is poor in oxygen and nutrient transport [10], we speculated that lumbar artery ligation in this animal model would further worsen the microenvironment of NP tissues.

Although T2-values of the ischemia group in our study tended to decrease compared to the control group, significance by two-way repeated measures ANOVA was not achieved. While significance might be obtained by increasing the number of animals, lumbar artery ligation would have only a mild effect on the matrix metabolism of the rabbit NP rather than inducing degenerative changes similar to human IVD degeneration.

Disc height narrowing, representing structural changes of IVDs, is a representative clinical sign of disc degeneration seen in lumbar radiographs. In this study, %DHI of the control and ischemia groups did not differ significantly throughout the observation period, indicating that ligation of lumbar arteries in this model did not affect to induce structural changes in corresponding IVDs. However, the %DHI of both the control and ischemia groups showed a slight, but significant, decrease from week 0 to 12. Vertebral heights at corresponding levels for measurement of DHI (L2-L6) continuously increased until 12 weeks after surgery (data not shown), while the disc height of corresponding IVDs did not increase significantly (data not shown). The discrepancy between the increase of vertebral and IVD height by growth is attributable to the relative decrease in %DHI in this rabbit model.

Safranin-O is a cationic dye composed of a mixture of dimethyl phenosafranin and trimethyl phenosafranin with a specific stoichiometric potential to bind glycosaminoglycans (GAGs) [31]. Safranin-O is, therefore, used for quantification and histological analysis of GAGs in articular cartilage and IVDs. The results of our study showed that staining patterns of Safranin-O in the NP changed after lumbar artery ligation, particularly in the pericellular area of corresponding IVDs, but not in AF tissues.

IVD cells, as well as chondrocytes, are surrounded by a narrow pericellular matrix (PCM) that plays a vital role in the biochemical and biomechanical properties of IVD cells [32–36]. The PCM is composed of a number of extracellular molecules, including a high concentration of the GAG-rich PG “aggrecan” [32]. In a three-dimensional alginate culture system, the PCM matrix metabolism is faster and more active than in other ECM regions [33–36]. Our histological observations of the rabbit NP suggest that matrix metabolism synthesis, especially aggrecan, would be stimulated in the PCM in response to ischemia in corresponding IVDs. Previous studies have shown that the mRNA expression of aggrecan by bovine NP cells was stimulated by hypoxia in vitro [37]. Furthermore, the expression of β-1,3-glucuronyltransferase 1 (GlcAt-1), a key enzyme in GAG synthesis, was increased under hypoxic conditions in vitro [38]. We evaluated the immunohistochemical expression of HIF-1α in the control and ischemia discs four weeks after surgery. Two-way ANOVA revealed that the ligation of lumbar arteries significantly affected a change in the percentage of HIF-1α immunoreactive cells of ischemia discs compared to that of control discs. Therefore, we speculated that ischemia of the lumbar spine produced by lumbar artery ligation would stimulate the synthesis of aggrecan, particularly in the NP of corresponding IVDs.

Total histological scores of cranial discs were significantly higher than those of control discs at 8 weeks after surgery; however no significant differences were identified between the control and ischemia group at other time points. Temporal changes in histological scores revealed that those scores had significantly increased at 4 and 8 weeks after ligation in the ischemia group, although no significant increases were identified at 12 weeks. This suggests that the histological changes following lumbar artery ligation were not progressive, but rather mild. Interestingly, the results of MRI analyses showed a significant inverse correlation between MRI T2-value and total histological score. When this correlation was independently evaluated for each category of the NP, the highest correlation coefficient was identified with the category of “NP pericellular matrix.” We speculated that the decrease in MRI T2-value in the ischemia groups would correspond with changes in the matrix integrity of the NP as evaluated by the staining properties of Safranin-O.

However, the matrix changes in the young rabbit NP were mild, and we found no progressive histological degenerative changes or structural changes that are found in human IVD degeneration. The rabbits at this age used in this study were skeletal immature [39], and those discs have a large number of notochordal cells which have great potential to regulate matrix metabolism including the synthesis of extracellular matrix [40–42]. Therefore, the author speculates that IVD tissues of young rabbits have the potential to resist and/or repair ischemic changes in the lumbar IVDs.

Clinically, degenerative disc diseases commonly develop in middle-aged or older human populations. In many cases, these populations already have systemic atherosclerosis and/or lumbar spondylosis changes, including IVD degeneration. There would be the possibility that impaired flow in lumbar arteries may enhance the degenerative changes of IVDs in middle-aged or aged-populations.

There are several limitations in this study. First, we did not perform arteriography to evaluate ischemia in the lumbar spines. Therefore, the extent of lumbar spine ischemia and the existence of collateral arteries to the corresponding vertebral levels remain unknown. The second limitation is that 12-week-old New Zealand White rabbits, roughly juvenile in human terms, were used in this study. Ideally, older rabbits should be used to evaluate the association between spinal ischemia and the disc degeneration found in human IVDs. The third limitation is that sham operation that only exposed the abdominal aorta was only used for a 0-week control group. Therefore, it cannot be denied the possibility that ligation of lumbar arteries influenced the blood flow of control (L2/3) disc at 4, 8, and 12 weeks after surgery. Forth, it has been reported that the response of rabbit IVDs against external stimuli such as disc puncture varies dependent on the disc level, probably due to the difference in disc volume [43]. Therefore, there is a possibility that the effect of diminishing flow on disc matrix changes in this animal model may differ between the upper and lower disc level. Lastly, it has been reported that T1 and T2 relaxation times were significantly influenced by the temperature of samples [44, 45]. Although the temperature of the rabbit IVD samples was not measured in this study, it would be great importance to measure the temperature of the samples and keep it constant during the MRI scan using a temperature probe in a temperature-controlled condition for accurately evaluating the T2-values of samples.

## Conclusions

In this study, we tested the hypothesis that ischemia of lumbar vertebrae initiates degenerative changes in corresponding IVDs. We used a rabbit lumbar artery ligation model. The results of this study reveal that the microenvironment, as evaluated by MRI T2-mapping and staining properties with Safranin-O, has in fact been changed in NP tissues after lumbar artery ligation. Further study using older rabbits is needed to evaluate the effect of impaired flow in lumbar arteries on degenerative changes in corresponding IVDs.

## Data Availability

The datasets used and/or analyzed during the current study available from the corresponding author on reasonable request.

## Abbreviations

AF: Annulus fibrosus
ANOVA: Analysis of variance
CC: Correlation coefficient
DHI: Disc height index
ECM: Extracellular matrix
GAGs: Glycosaminoglycans
GLUT1: Glucose transporters 1
HIF: Hypoxia inducible factor
IVD: Intervertebral disc
MRI: Magnetic resonance imaging
NP: Nucleus pulposus
PCM: Pericellular matrix
PGs: Proteoglycans
ROI: Regions of interest
TE: Time-to-Echo
TR: Time-to-Repeat
